# Distinct Mechanisms Underlie Developmental Plasticity and Adult Acclimation of Thermogenic Capacity in High-Altitude Deer Mice

**DOI:** 10.3389/fphys.2021.718163

**Published:** 2021-08-11

**Authors:** Catherine M. Ivy, Haley Prest, Claire M. West, Graham R. Scott

**Affiliations:** Department of Biology, McMaster University, Hamilton, ON, Canada

**Keywords:** high-altitude adaptation, aerobic performance, oxygen transport, breathing, lung structure, hematology

## Abstract

Developmental plasticity can elicit phenotypic adjustments that help organisms cope with environmental change, but the relationship between developmental plasticity and plasticity in adult life (e.g., acclimation) remains unresolved. We sought to examine developmental plasticity and adult acclimation in response to hypoxia of aerobic capacity (V̇O_2max_) for thermogenesis in deer mice (*Peromyscus maniculatus*) native to high altitude. Deer mice were bred in captivity and exposed to normoxia or one of four hypoxia treatments (12 kPa O_2_) across life stages: adult hypoxia (6–8 weeks), post-natal hypoxia (birth to adulthood), life-long hypoxia (before conception to adulthood), and parental hypoxia (mice conceived and raised in normoxia, but parents previously exposed to hypoxia). Hypoxia during perinatal development increased V̇O_2max_ by a much greater magnitude than adult hypoxia. The amplified effect of developmental hypoxia resulted from physiological plasticity that did not occur with adult hypoxia – namely, increases in lung ventilation and volume. Evolved characteristics of deer mice enabled developmental plasticity, because white-footed mice (*P. leucopus*; a congener restricted to low altitudes) could not raise pups in hypoxia. Parental hypoxia had no persistent effects on V̇O_2max_. Therefore, developmental plasticity can have much stronger phenotypic effects and can manifest from distinct physiological mechanisms from adult acclimation.

## Introduction

Developmental plasticity is the process by which phenotypes are altered by the early life environment, and is often viewed to be irreversible and underpinned by distinct mechanisms from phenotypic plasticity in adult life (e.g., acclimation; Burggren and Reyna, 2011; Moczek et al., 2011; Burggren, 2020). The phenotypic responses to conditions experienced during development are not always the same as responses during adult life, and responses can even vary between developmental stages (e.g., prenatal vs. postnatal development; Carroll, 2003; Bavis, 2005; Ho and Burggren, 2012; West et al., 2021a). However, emerging evidence is challenging the distinction between developmental plasticity and adult acclimation, suggesting that developmental plasticity can be reversible and linked to plasticity in later life (Scott and Johnston, 2012; Beaman et al., 2016; Slotsbo et al., 2016; Burggren, 2020). However, for many performance traits that are critical to fitness, we are just beginning to understand the importance and life-stage specificity of developmental plasticity, and how it might differ from reversible acclimation during adulthood.

Small mammals at high altitude are well suited to examining plasticity during development and adulthood. The cold and oxygen-depleted (hypoxic) environment at high altitude requires that endotherms sustain high rates of O_2_ consumption for heat generation (thermogenesis) and locomotion, while facing a diminished O_2_ supply. This is particularly challenging for mammals of small body size, which face greater demands for thermogenesis as a result of a larger surface area to volume ratio. As a result, some small mammals at high altitude have evolved increased aerobic capacity for thermogenesis or exercise [quantified as maximal O_2_ consumption (V̇O_2max_) during acute cold exposure or intense running] in hypoxia (McClelland et al., 1998; Chappell et al., 2007; Schippers et al., 2012; Lui et al., 2015; Tate et al., 2017, 2020). Hypoxia-induced plasticity can also contribute to increasing V̇O_2max_ at high altitude (Storz et al., 2010b; Ivy and Scott, 2015). In humans, for example, developmental exposure to high-altitude hypoxia increases aerobic capacity in hypoxia (Sun et al., 1990; Frisancho et al., 1995; Brutsaert et al., 1999; Kiyamu et al., 2015a; Brutsaert, 2016). However, few previous studies have distinguished the effects of hypoxia at different life stages, such as between pre-natal and early post-natal development. These distinctions may be quite important in light of critical windows during early post-natal life for lung morphogenesis (Burri, 1984; Massaro and Massaro, 2002; Frappell and MacFarlane, 2006) and for the development of neural networks that control breathing (Wong-Riley et al., 2019). Furthermore, because high-altitude hypoxia is unremitting and unavoidable throughout life, the effects of hypoxia at early life stages might influence responses to hypoxia during adulthood. Small mammals that are native to high altitude thus provide an opportunity to examine how environmental effects across multiple distinct life stages – both early developmental and adult – might interactively affect complex adult phenotypes.

The objective of this study was to gain insight into these issues by investigating the influence of developmental plasticity and adult acclimation in hypoxia on thermogenic V̇O_2max_ in deer mice (*Peromyscus maniculatus*) native to high altitude. Deer mice are broadly distributed across North America and can be found from sea level to over 4,300 m elevation in the Rocky Mountains (Hock, 1964; Snyder et al., 1982; Natarajan et al., 2015). Adults from wild populations at high altitude sustain higher metabolic rates than those at low altitude (Hayes, 1989), likely to support the increased demands of thermogenesis in cold alpine environments. There can be strong directional selection for increased thermogenic V̇O_2max_ at high altitude (Hayes and O’Connor, 1999), which has led to evolved increases in thermogenic V̇O_2max_ in hypoxia in high-altitude deer mice compared to low-altitude deer mice and white-footed mice (*P. leucopus*, a congeneric species that is restricted to low altitudes; Cheviron et al., 2012, 2013, 2014; Lui et al., 2015; Tate et al., 2017, 2020). Differences in thermogenic V̇O_2max_ and in various respiratory and metabolic traits that underlie it become apparent ~2–3 weeks after birth in comparisons between high- and low-altitude mice raised in normoxia (Robertson et al., 2019; Robertson and McClelland, 2019; Ivy et al., 2020; West et al., 2021a). However, although the effects of adult acclimation to hypoxia on thermogenic V̇O_2max_ and its underlying determinants have been described (Lui et al., 2015; Lau et al., 2017; Tate et al., 2017, 2020), and life-long exposure to high altitude has been shown to increase thermogenic V̇O_2max_ (Chappell et al., 2007), the specific effects of hypoxia exposure during pre-natal and post-natal development has not been resolved. The current study seeks to address these knowledge gaps. We test the hypothesis that developmental hypoxia elicits a greater increase in thermogenic V̇O_2max_ and acts *via* distinct mechanisms as compared to hypoxia acclimation during adulthood.

## Materials and Methods

### Mouse Populations and Hypoxia Exposures

Captive breeding populations were established from wild populations of deer mice native to high altitude near the summit of Mount Evans, CO, United States (39°35'18"N, 105°38'38"W; 4,350 m above sea level; *P. m. rufinus*) and white-footed mice (*P. leucopus*; a species that is restricted to low altitudes) native to the Great Plains (Nine Mile Prairie, Lancaster County, NE, United States, at 40°52'12"N, 96°48'20.3"W, 430 m above sea level). Occupation of low-altitude habitats is the ancestral condition for the phylogenetic clade containing *Peromyscus maniculatus* and *P. leucopus*, and *P. leucopus* is a good representative of this ancestral condition from which *P. maniculatus* evolved and expanded their elevational range (Velotta et al., 2018). Wild adults were transported to McMaster University (~50 m above sea level) and housed in common laboratory conditions, and were used as parental stock to produce first generation (G1) lab progeny for each mouse population. Breeding pairs were held in individual cages, the male was removed when the female was visibly pregnant, and pups were weaned and moved to separate cages at post-natal day (P)21. G1 mice were similarly used as parental stock to produce second generation (G2) progeny for each population. Experiments were conducted on three distinct families of adult G2 mice for each population. All mice were held at 24–25°C and a photoperiod of 12 h light: 12 h dark, and were provided with unlimited access to standard rodent chow and water. All animal protocols followed guidelines established by the Canadian Council on Animal Care and were approved by the McMaster University Animal Research Ethics Board.

**Figure 1:**
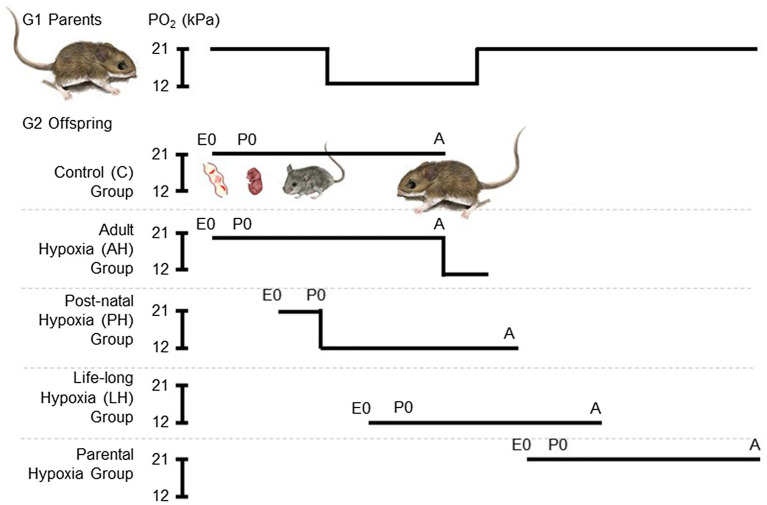
Experimental treatment groups used to evaluate the effects of chronic hypoxia at different life stages. We used a standardized breeding design with first generation (G1) lab-raised parents to expose second generation (G2) lab-raised offspring to hypoxia starting at multiple life stages. Each breeding pair conceived and raised their 5th and 6th litters in normobaric normoxia until weaning. These progeny were split into two treatment groups, one that remained in normoxia (~21 kPa O_2_; control group, C) and the other that was acclimated to hypobaric hypoxia (~12 kPa O_2_) for 6–8 weeks during adulthood (adult hypoxia group, AH). Litter 7 was conceived and born in normoxia, but parents and offspring were moved to hypobaric hypoxia within 12 h of birth, and offspring were weaned and raised to adulthood in hypobaric hypoxia (post-natal hypoxia group, PH). The mother and father continued to be held in hypobaric hypoxia, and were allowed to conceive litter 8, which was born and raised into adulthood in hypobaric hypoxia (life-long hypoxia group, LH). After weaning litter 8, breeding pairs were returned to normoxia and were then allowed to conceive and raise litter 9 in normoxia (parental hypoxia group). Full details of the breeding design can be found in the Methods. E, embryonic age in days; P, post-natal age in days; A, adult; and PO_2_, partial pressure of O_2_.

We used a standardized breeding design to expose G2 mice to hypoxia, starting at a range of different life stages, with five different treatment groups (Figure 1), as previously described (Nikel et al., 2018). Each breeding pair was first allowed to raise 4 litters, in order to avoid potential effects of variation in litter size and resource allocation that may arise across the first few litters (Kirkland and Layne, 1989). Each pair then conceived and raised litters 5 and 6 in standard cage conditions of normobaric normoxia until weaning. These progeny from each family were split into two treatment groups, one that remained in normoxia (control group, C) and the other that was acclimated to hypobaric hypoxia (barometric pressure of 60 kPa, ~12 kPa O_2_; simulating the hypoxia at an elevation of 4,300 m) for 6–8 weeks during adulthood (adult hypoxia group, AH). Litter 7 was also conceived and born in normoxia, but the family was moved to hypobaric hypoxia within 12 h of birth, and the mother and pups remained there together until weaning. After weaning, litter 7 pups continued to be raised in hypobaric hypoxia into adulthood (post-natal hypoxia group, PH). The mother and father continued to be held in hypobaric hypoxia, and were allowed to conceive litter 8, which was born and raised into adulthood in hypobaric hypoxia (life-long hypoxia group, LH). After weaning litter 8, breeding pairs were returned to normoxia and were then allowed to conceive and raise litter 9 in normoxia (parental hypoxia group). Litter sizes that survived to weaning for highlanders across treatment groups: litters 5 and 6 used for C and AH groups, 4–6 pups per litter, 13 males and 13 females in total; litter 7 for PH group, three pups per litter, two males and seven females in total; litter 8 for LH group, 2–4 pups per litter, six males and three females in total; litter 9 for parental hypoxia group, 2–3 pups per litter, three males and four females in total. Litter sizes that survived to weaning for lowlanders: litters 5 and 6, 5–7 pups per litter, 21 males and 13 females in total; litter 7, 0 pups; litter 8, 0 pups; litter 9: three pups per litter, two males and four females in total (in lowlanders, litter 9 was only conducted in two families owing to temporary suspension of research activities during the COVID-19 pandemic). Exposures to hypobaric hypoxia were conducted using specially designed hypobaric chambers (McClelland et al., 1998; Lui et al., 2015). Cages were cleaned twice a week during hypoxia exposures, which required that mice be returned to normobaria for a brief period (<20 min).

### Measurements of Thermogenic V̇O_2max_ and Cardiorespiratory Physiology

Thermogenic V̇O_2max_ was measured between 6 and 8 months of age along with concurrent measurements of breathing, arterial O_2_ saturation (SaO_2_), and heart rate. Thermogenic V̇O_2max_ was measured using open-flow respirometry as the maximal rate of O_2_ consumption achieved over 30 s during a 10 min exposure to acute cold (−5°C) in a heliox gas mixture containing both normoxic (21 kPa O_2_, balance He) and hypoxic (12 kPa O_2_, balance He) O_2_ levels. Normoxic and hypoxic trials were conducted in randomized order and separated by at least 48 h. Measurements were conducted in a custom-made apparatus that allowed for simultaneous measurements of breathing by plethysmography as well as SaO_2_ and heart rate by pulse oximetry, as previously described (Tate et al., 2020). The apparatus consisted of a 530 ml respirometry chamber, in which the animal was placed, and an empty reference chamber. The respirometry chamber contained a pneumotachograph that provided a natural leak to the external environment, allowing for changes in flow relative to the reference chamber (caused by breathing) to be detected using a differential pressure transducer (Validyne DP103-18; Cancoppas, Mississauga, ON, Canada). The chamber also contained ports for incurrent and excurrent gas flows and ports for leads from the pulse oximetry collar. During measurements, heliox inflow was delivered to the respirometry chamber at 1500 ml min^−1^_,_ regulated using a mass flow controller (MFC-2, Sable Systems, Las Vegas, NV, United States) and precision flow control valves for oxygen and helium (Sierra Instruments, Monterey, CA, United States), and excurrent gas leaving the respirometry chamber was subsampled at 200 ml min^−1^, dried with pre-baked Drierite, and analyzed for O_2_ and CO_2_ fractions (FoxBox Respirometry System, Sable Systems). The entire apparatus was held within a freezer to maintain the respirometry chamber at or slightly below −5°C (verified with a PT-6 thermocouple; Physitemp, Clifton, NJ, United States).

Thermogenic V̇O_2max_ trials were conducted as previously described (Tate et al., 2020). Before the start of each trial, the differential pressure transducer was calibrated, while the heliox gas mixture flowed through the empty respirometry chamber, by withdrawing and reinjecting 200 μl of gas from the respirometry chamber multiple times using a Hamilton syringe (at a flowrate similar to a mouse breathing). Baseline O_2_ and CO_2_ fractions were then measured, without an animal in the respirometry chamber. At the start of each trial, mice were weighed and instrumented with a collar for a MouseOx Plus pulse oximeter (Starr Life Sciences, PA, United States), which required the fur to be removed from a small area on the neck (3 days in advance). Mice were habituated to the pulse oximeter collars 1 day in advance of thermogenic trials. Mice were then placed inside the respirometry chamber for 10 min, during which time, we measured O_2_ and CO_2_ fractions of excurrent gas leaving the chamber, the changes in flow across the pneumotachograph that were caused by ventilation, and the pulse oximetry signals. Mice were then removed from the chamber, core body temperature was measured immediately using a mouse rectal probe (RET-3-ISO; Physitemp), and mice were finally returned to their cage in the appropriate acclimation environment. All mice had depressed core body temperature at the end of the acute cold exposure. In some cases, mice removed the pulse oximetry collar during measurement; therefore, the trial was repeated after a minimum of 48 h recovery.

Thermogenic V̇O_2max_ was defined as the highest O_2_ consumption rate achieved over a 30 s period during the trial (generally occurring after ~ 4–6 min in the chamber), and was calculated using established formulas for baseline and excurrent measurements of O_2_ and CO_2_ fractions (Lighton, 2008).

$$\dot{V}O_{2\max}=\left[F_{iO_{2}}-\left(\frac{\left(1-F_{iO_{2}}-F_{iCO_{2}}\right)}{\left(1-F_{eO_{2}}-F_{eCO_{2}}\right)}\right)F_{eO_{2}}\right]\times FR_{i}$$

Where *F_i_* and *F_e_* denote incurrent and excurrent fraction and *FR_i_* denotes incurrent flow rate. Breathing, SaO_2_, and heart rate were then determined at V̇O_2max_. Tidal volume was determined using the barometric method of whole-body plethysmography, calculated from the flows that were measured across the pneumotachograph using established equations for flow-through respirometry (Drorbaugh and Fenn, 1955; Jacky, 1980). Total ventilation was the product of tidal volume and breathing frequency. Air convection requirement is the quotient of total ventilation and V̇O_2max_, and pulmonary O_2_ extraction was calculated as V̇O_2max_ divided by the product of total ventilation and the O_2_ concentration of inspired air. All of the above data were acquired using a PowerLab 8/32 and Labchart 8 Pro software (ADInstruments, Colorado Springs, CO, United States). Pulse oximetry measurements of SaO_2_ and heart rate were recorded using Starr Life Sciences acquisition software.

### Lung Volume and Histology

Following blood collection and careful excision of the heart, lung volume was measured on a subset of mice in the N, AH, and LH groups. The trachea was intubated with PE50 tubing (BD Intramedic, FisherScientific, Mississauga, ON, Canada) and secured with 2-0-gage suture silk (Prolene, FisherScientific). The lungs were then inflated with 10% formalin at a pressure of 30 cmH_2_O (Limjunyawong et al., 2015). The trachea was then tied closed and the lungs were carefully excised from the body cavity. Lung volume was measured immediately using the immersion displacement technique (Scherle, 1970). Lungs were then immersed in 10% formalin and fixed for 72 h, then stored in 70% ethanol until paraffin embedding. Embedded lungs were sectioned using a microtome at a thickness of 5 μm and sections were mounted on Superfrost Plus microscope slides (Fisher Scientific; Mississauga, ON, Canada). Sections were taken at each of 3–4 different locations along the rostrocaudal axis of both the left and right lungs. Sections were then stained for hematoxylin and eosin as follows. Sections were deparaffinized with two washes of xylene for 10 min each, incubated in two changes of 100% ethanol and one of 95% ethanol for 5 min each. Sections were washed in distilled water for 5 min, stained with Gills II hematoxylin for 2 min, washed in water for 1 min, and stained with eosin for 45 s. Sections were then rinsed in water and dehydrated using one wash of 95% ethanol and two washes of 100% ethanol for 5 min each. Sections were cleared with two changes of xylene for 10 min each, then coverslipped with Permount (Fisher Scientific).

Stained sections were imaged for analysis using an upright brightfield microscope. Images were taken at 200X magnification from three various regions within each section, yielding 12–18 images for analysis per individual. Stereological methods were then used to make unbiased morphometric measurements of alveolar surface density (surface area per volume of lung parenchyma) and the total alveolar surface area of the animal, as previously described (Mühlfeld et al., 2012; West et al., 2021a). Alveolar density was quantified as the number of distinct alveoli per imaged area of lung parenchyma.

### Statistics

Linear mixed-effects models were used to test for the main effect of hypoxia treatment within each mouse population (highland and lowland) using the lme4 package in R (v. 3.6.0; Bates et al., 2015). We used a backwards model selection approach, in which initial models included body mass as a covariate and sex and family as random effects. If these terms had *p*-values above 0.1, they were removed by stepwise backward deletion (starting with the term with the highest *p* value) and the model was re-run until all random effects or covariates in the model had *p* values below 0.1. In most cases, family and sex were not significant and were thus excluded from most final models. Statistical analyses were carried out on absolute values of traits that were not corrected for body mass (because effects of body mass were accounted for in statistical models), but the V̇O_2max_ and ventilatory volume data presented here are expressed relative to body mass as is conventional in the literature. The full results of statistical models are included in the electronic supplementary material (Supplementary Tables S1 and S2), and the salient findings are reported in the Results. Holm-Sidak post-tests were used as appropriate to make pairwise comparisons when significant effects of hypoxia treatment were detected. A value of *p* < 0.05 was considered to be significant.

## Results

### Thermogenic V̇O_2max_

**Figure 2:**
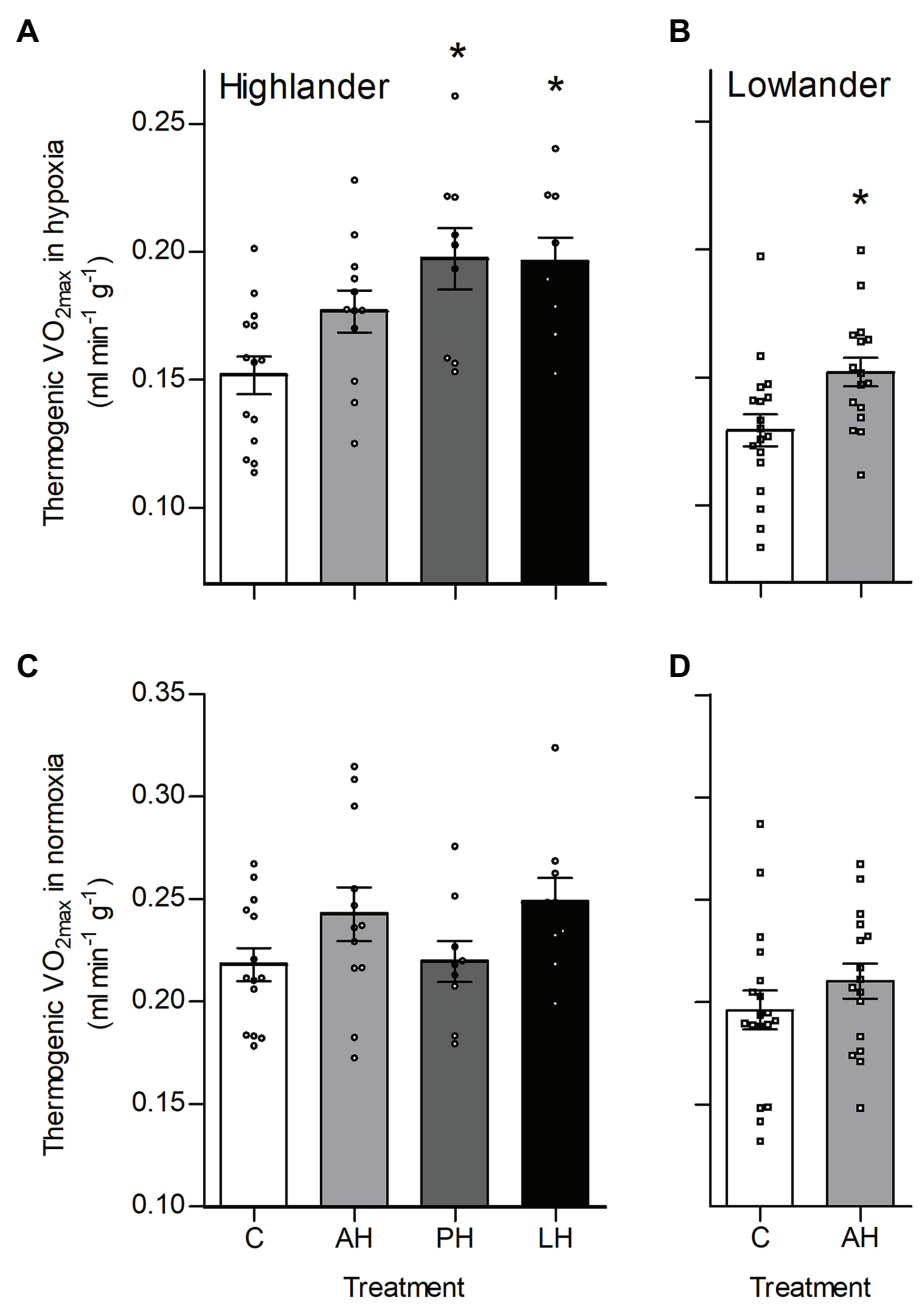
Developmental hypoxia increased thermogenic capacity in hypoxia in deer mice native to high altitude. Thermogenic capacity was measured as the maximal rate of O_2_ consumption (V̇O_2max_) during acute cold (−5°C) exposure in hypoxic heliox (~12 kPa O_2_; **A,B**) and normoxic heliox (~21 kPa O_2_; **C,D**). Lowland mice were unable to raise litters in the PH or LH groups. Bars indicate mean ± SEM and symbols represent individual values. Treatment groups are shown in Figure 1 with N as follows: 14 control (C), 12 AH, nine PH, and nine LH for highlanders; 18 C and 16 AH for lowlanders. ^*^Significant pairwise difference from control within each population using Holm-Sidak post-tests.

Hypoxia during early development enhanced thermogenic V̇O_2max_ in hypoxia by a greater magnitude than hypoxia acclimation during adulthood (Figure 2). Specifically, thermogenic V̇O_2max_ of highland deer mice was ~28–33% greater in post-natal hypoxia and life-long hypoxia groups compared to controls raised and held in normoxia, whereas the adult hypoxia was only ~16% greater than normoxic controls on average (Figure 2A). Lowland white-footed mice did not survive to weaning in the post-natal and life-long hypoxia groups (some pups were born but typically died 10–20 days after birth), but hypoxia acclimation in adulthood increased thermogenic V̇O_2max_ in hypoxia compared to controls (Figure 2B). In contrast, thermogenic V̇O_2max_ in normoxia was not influenced by hypoxia treatment in either highland or lowland mice (Figures 2C,D). Parental hypoxia had no effects on thermogenic V̇O_2max_ in hypoxia or normoxia in either population (Supplementary Table S3).

**Figure 3:**
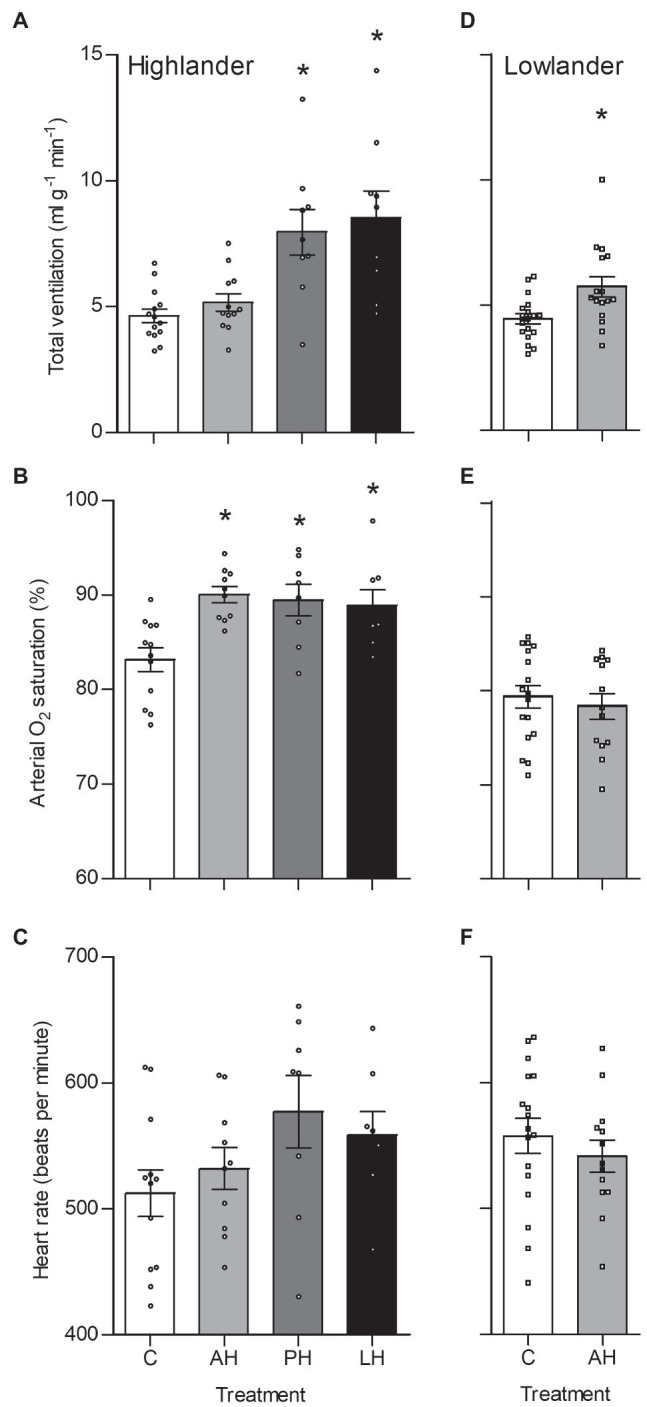
Total ventilation (A,D), arterial O_2_ saturation (SaO_2_) (B,E), and heart rate (C,F) measured at thermogenic V̇O_2max_ in hypoxia in highland (A,B,C) and lowland (D,E,F) mice. Lowland mice were unable to raise litters in the PH or LH groups. Bars indicate mean ± SEM and symbols represent individual values. Treatment groups are shown in Figure 1 with N as follows: 14 control (C), 12 acute hypoxia (AH), 9 post-natal hypoxia (PH), and 9 life-long hypoxia (LH) for highlanders; 18 C and 16 AH for lowlanders. *Significant pairwise difference from control within each population using Holm-Sidak post-tests.

**Figure 4:**
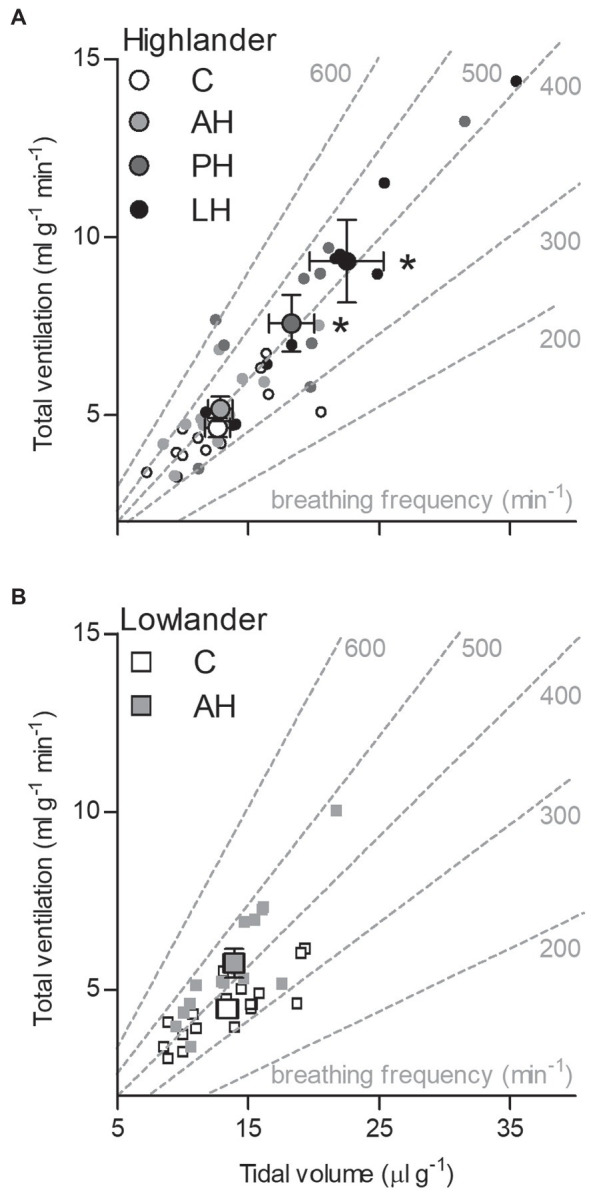
The effects of developmental hypoxia on total ventilation in highland deer mice (A) arose from increases in maximal tidal volume, with no change in breathing frequency, in contrast to lowland mice (B). Gray dashed lines represent isopleths of constant breathing frequency. Large symbols with error bars indicate mean ± SEM and small symbols represent individual values. Lowland mice were unable to raise litters in the PH or LH groups. Bars indicate mean ± SEM and symbols represent individual values. Treatment groups are shown in Figure 1 with N as follows: 14 control (C), 12 acute hypoxia (AH), 9 post-natal hypoxia (PH), and 9 life-long hypoxia (LH) for highlanders; 18 C and 16 AH for lowlanders. *Significant pairwise difference from control within each population using Holm-Sidak post-tests.

The effects of developmental hypoxia in highlanders on thermogenic V̇O_2max_ in hypoxia were associated with increases in total ventilation (Figure 3). Total ventilation was significantly higher in post-natal hypoxia (~63%) and life-long hypoxia (~100%) groups compared to normoxic controls, but total ventilation was unaltered in the adult hypoxia group (Figure 3A). The magnitude of variation in total ventilation equaled or exceeded that of V̇O_2max_ in hypoxia, as reflected by an increase in air convection requirement in the life-long hypoxia group (Supplementary Tables S1 and S4). The increases in total ventilation with developmental hypoxia were driven by deeper tidal volumes but no significant changes in breathing frequency (treatment effect, *p* = 0.428; Supplementary Tables S1 and S4). This was reflected by the shift toward the top-right along isopleths of constant breathing frequency in plots of total ventilation vs. tidal volume (Figure 4A). Arterial O_2_ saturation in hypoxia was ~83% in controls, but was significantly greater at ~90% in all hypoxia treatment groups (adult and developmental; Figure 3B). In contrast, there was no significant treatment effect on heart rate at thermogenic V̇O_2max_ in hypoxia (*p* = 0.147; Figure 3C). Ventilation, arterial O_2_ saturation, and heart rate at thermogenic V̇O_2max_ in normoxia were not altered with developmental hypoxia treatments (Table 1). In contrast, whereas lowland mice exhibited a modest increase in total ventilation after adult hypoxia acclimation compared to normoxic controls (Figure 3D), this change was driven by higher breathing frequency (treatment effect, *p* < 0.001) and no change in tidal volume (treatment effect, *p* = 0.977; Figure 4B). Lowland mice acclimated to chronic hypoxia in adulthood did not increase arterial O_2_ saturation (treatment, *p* = 0.472) or heart rate (treatment, *p* = 0.417) at thermogenic V̇O_2max_ in hypoxia (Figures 3E,F). During thermogenic V̇O_2max_ in normoxia, lowland mice exhibited similar increases in breathing as observed during thermogenic V̇O_2max_ in hypoxia (Table 1; Supplementary Table S4). Interestingly, despite the observation that parental hypoxia had no effects on thermogenic V̇O_2max_, highland mice in the parental hypoxia group had higher arterial O_2_ saturation at hypoxic V̇O_2max_ (90.03 ± 1.95%) compared to normoxic mice, similar to the increases observed in developmental and adult hypoxia groups. However, parental hypoxia had no other effects in highlanders, and did not affect total ventilation, arterial O_2_ saturation, or heart rate in lowlanders (Supplementary Table S3).

**Table 1:** Measurements at thermogenic V̇O_2max_ at 21 kPa O_2_ for highland deer mice and lowland white-footed mice exposed to hypoxia during different stages of development.

Trait	Treatment	Highlander	Lowlander
Arterial O_2_ saturation (%)	Control	98.82 ± 0.35	97.75 ± 0.50
	Adult hypoxia	97.46 ± 1.49	97.94 ± 0.45
	Post-natal hypoxia	99.26 ± 0.13	-
	Life-long hypoxia	99.41 ± 0.15	-
Heart rate (beats min^−1^)	Control	628.3 ± 17.3	640.8 ± 15.8
	Adult hypoxia	631.5 ± 21.9	655.9 ± 17.1
	Post-natal hypoxia	635.2 ± 28.4	-
	Life-long hypoxia	630.6 ± 36.8	-
Total ventilation (ml min^−1^ g^−1^)	Control	5.55 ± 0.93	4.57 ± 0.24
	Adult hypoxia	4.91 ± 0.23	5.35 ± 0.20^*^
	Post-natal hypoxia	5.94 ± 0.62	-
	Life-long hypoxia	7.76 ± 0.92	-
Tidal volume (μl g^−1^)	Control	13.97 ± 2.58	12.80 ± 0.84
	Adult hypoxia	11.27 ± 0.69	13.37 ± 0.59
	Post-natal hypoxia	15.36 ± 1.64	-
	Life-long hypoxia	17.97 ± 1.12	-

*Data are mean ± SEM, with N as in*Figure 2.

* *Significant pairwise difference from control within a population using Holm-Sidak post-tests*.

### Lung Structure

**Figure 5:**
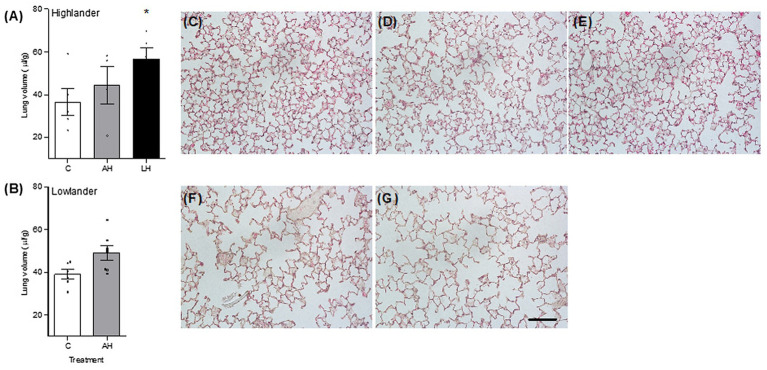
Developmental hypoxia increased lung volume **(A,B)**. Lung parenchyma structure was preserved between control (C; **C,F**), adult hypoxia (AH; **D,G**), and life-long hypoxia (LH; **E**) treatment groups in highlanders **(C–E)** and lowlanders **(F,G)**. Bars indicate mean ± SEM and symbols represent individual values. Treatment groups are shown in Figure 1 with N as follows: five C, four AH, and five LH for highlanders; six C and seven AH for lowlanders. Each image has the same scale, and the scale bar represents 100 μm. Lung morphometrics are reported in Table 2. *Significant pairwise difference from control within each population using Holm-Sidak post-tests.

Developmental hypoxia increased lung volume, but hypoxia treatment had minimal influence on other aspects of lung morphology (Figure 5; Table 2; Supplementary Table S5). In highlanders, lung volume increased by ~54% on average in the life-long hypoxia group compared to controls, but effects of adult hypoxia were not significant (Figure 5A). There were no significant changes in mean alveolar surface density (treatment effect, *p* = 0.406), total alveolar surface area (*p* = 0.276), or alveolar density (*p* = 0.608) between treatment groups in highlanders. In lowlanders, effects of adult hypoxia acclimation on lung volume were not significant (treatment effect, *p* = 0.066; Figure 5B), nor were there any significant changes in mean alveolar surface density (*p* = 0.657) or total alveolar surface area (*p* = 0.069).

**Table 2:** Histological measurements of lung morphology.

Trait	Treatment	Highlander	Lowlander
Alveolar surface density (μm^−1^)	Control	0.108 ± 0.003	0.089 ± 0.003
	Adult hypoxia	0.102 ± 0.002	0.091 ± 0.102
	Life-long hypoxia	0.099 ± 0.006	-
Total alveolar surface area (cm^2^ g^−1^)	Control	31.44 ± 5.26	27.78 ± 2.03
	Adult hypoxia	35.81 ± 6.59	35.68 ± 2.62
	Life-long hypoxia	44.18 ± 5.11	-
Alveolar density (mm^−2^)	Control	885 ± 51	770 ± 27
	Adult hypoxia	853 ± 86	661 ± 30^*^
	Life-long hypoxia	815 ± 73	-

*Data are mean ± SEM. N as follows: five control, four adult hypoxia, and five life-long hypoxia for highlanders; six control and seven adult hypoxia for lowlanders*.

* *Significant pairwise difference from control within a population using Holm-Sidak post-tests*.

### Hematology and Right-Ventricle Hypertrophy

The effects of developmental hypoxia on hematology were similar to the effects of adult hypoxia, but developmental hypoxia had some unique effects on the right ventricle (Table 3; Supplementary Table S6). In highlanders, hematocrit increased similarly among hypoxia treatment groups (adult and developmental) compared to controls. This likely reflected erythrocyte swelling, because there were no changes in blood hemoglobin content (treatment effect, *p* = 0.987) and significant decreases in mean corpuscular hemoglobin content compared to controls. In lowlanders, adult hypoxia increased hematocrit, blood hemoglobin content, and mean corpuscular hemoglobin content, suggesting that erythropoiesis and/or relative reductions in plasma volume were more important contributors to hematological changes than erythrocyte swelling. Right-ventricle hypertrophy did not occur in highlanders during adult hypoxia acclimation, in contrast to the robust hypertrophy that occurred in lowlanders, but hypertrophy did occur in highlanders after developmental hypoxia treatment. Parental hypoxia had no effect on hematology or right-ventricle hypertrophy in highlanders, but in lowlanders it increased blood hemoglobin content (30.0 ± 1.1 g dl^−1^) and mean corpuscular hemoglobin content (67.3 ± 3.1 g dl^−1^), and it increased the relative mass of the right ventricle (0.32 ± 0.02; Supplementary Table S6).

**Table 3:** Tissue measurements in highland deer mice and lowland white-footed mice exposed to hypoxia.

Trait	Treatment	Highlander	Lowlander
Blood hemoglobin content (g dl^−1^)	Control	18.96 ± 1.11	15.30 ± 0.43
	Adult hypoxia	18.64 ± 0.42	25.08 ± 2.10^*^
	Post-natal hypoxia	18.67 ± 0.59	-
	Life-long hypoxia	19.04 ± 1.32	-
Hematocrit (%)	Control	46.48 ± 0.65	46.22 ± 0.77
	Adult hypoxia	54.62 ± 1.19^*^	56.79 ± 1.06*^*^
	Post-natal hypoxia	53.67 ± 1.28^*^	-
	Life-long hypoxia	58.77 ± 1.31^*^	-
Mean corpuscular hemoglobin content (g dl^−1^)	Control	40.72 ± 2.14	33.12 ± 0.79
	Adult hypoxia	34.30 ± 1.10^*^	44.61 ± 4.16^*^
	Post-natal hypoxia	34.44 ± 1.12^*^	-
	Life-long hypoxia	32.58 ± 2.59^*^	-
Right ventricle mass relative to left ventricle and septum mass	Control	0.21 ± 0.01	0.25 ± 0.01
	Adult hypoxia	0.23 ± 0.02	0.34 ± 0.02^*^
	Post-natal hypoxia	0.30 ± 0.02^*^	-
	Life-long hypoxia	0.31 ± 0.03^*^	-

*Data is mean ± SEM, with N as in*Figure 2.

* *Significant pairwise difference from control within a population*.

## Discussion

Developmental plasticity can help organisms respond to environmental change, but for many performance traits that are critical to fitness, we still have a poor understanding of the importance and life-stage specificity of developmental plasticity and how it relates to reversible acclimation during adulthood. Here, we show that high-altitude deer mice exposed to hypoxia during early development had augmented thermogenic capacity in hypoxia later in adult life, and the effect appeared to be larger when hypoxia exposure began at earlier life stages (i.e., pre-natal vs. post-natal life). The magnitude of developmental plasticity was much greater than the effects of hypoxia acclimation during adulthood, and it arose *via* distinct mechanisms (increases in lung ventilation and volumes). This parallels recent findings that developmental hypoxia leads to effects on resting breathing pattern that are not elicited by hypoxia acclimation in high-altitude deer mice (Ivy and Scott, 2017, 2021). Evolved characteristics of deer mice enabled developmental plasticity in hypoxia, because lowland white-footed mice could not successfully raise young in hypoxia. In light of the advantage of a high thermogenic V̇O_2max_ for survival (Hayes and O’Connor, 1999), developmental plasticity in hypoxia is likely adaptive and may improve fitness in deer mice native to high altitude.

Our finding that neonatal and life-long hypoxia enhanced V̇O_2max_ in hypoxia but not in normoxia suggests that developmental hypoxia reduced the depressive effects of hypoxia on aerobic capacity, consistent with studies in humans with high-altitude ancestry (Frisancho et al., 1973, 1995; Greksa et al., 1985; Sun et al., 1990; Brutsaert et al., 1999; Kiyamu et al., 2015a; Brutsaert, 2016). For example, among Peruvian Quechua, individuals that were born and raised to adulthood at high altitude had significantly higher aerobic capacity during exercise in hypoxia than individuals that were born and raised at sea level, in association with increased vital capacity and arterial O_2_ saturation during exercise (Kiyamu et al., 2015a,b). However, the magnitude of these developmental effects in humans (≤10%) have often been smaller than those observed here in deer mice (≥30%). Unlike deer mice, developmental hypoxia often reduces absolute ventilation and/or ventilation relative to O_2_ consumption (i.e., air convection requirement) during exercise in adult humans (Brutsaert, 2016). By increasing both total ventilation and lung volume, developmental hypoxia in deer mice may elicit greater increases in thermogenic V̇O_2max_ in hypoxia than would be achieved by increasing lung volume alone. This could arise as increased total ventilation (*via* tidal volume) augments alveolar ventilation, and thus the alveolar O_2_ tension driving diffusion, while increased lung volume combined with preserved alveolar surface density augments pulmonary O_2_ diffusing capacity.

The expansion of lung volume and preservation of alveolar surface density with developmental hypoxia suggests that highland deer mice overcome the detrimental effects of hypoxia during critical windows of lung morphogenesis during early post-natal life. The formation of lung alveoli occurs during early post-natal life in mice, and hypoxia exposure during this period can impede lung development by impairing septation in lowland mice (Blanco et al., 1991; Ambalavanan et al., 2008). However, we have previously shown that highland mice exposed to developmental hypoxia suffer no impairment in alveolar formation from 7 to 30 days after birth, and exhibit increased lung volume from 14 to 30 days (West et al., 2021a). Our findings here show that these patterns persist into adulthood. Similarly, colonies of domestic mice bred for many generations in captivity at 3600 m in La Paz, Bolivia exhibit increased lung volume and alveolar surface area in adulthood compared to sea level controls (Jochmans-Lemoine et al., 2018). Avoidance of the pathological effects of hypoxia on early lung development is likely critical for maintaining high pulmonary O_2_ diffusing capacity and contributing to increased thermogenic V̇O_2max_ in high-altitude mice.

The increases in pulmonary ventilation and volumes induced by developmental hypoxia likely interact with evolved and acclimation-induced variation in other traits across the O_2_ transport pathway in high-altitude deer mice. Our findings here are consistent with previous observations that highlanders exhibit higher arterial O_2_ saturation at V̇O_2max_ than lowlanders, which likely arise from population differences in pulmonary function and hemoglobin-O_2_ affinity (Snyder et al., 1982; Storz et al., 2009, 2010a; Ivy et al., 2020; Tate et al., 2020; West et al., 2021a,b). Developmental hypoxia increases hemoglobin-O_2_ affinity starting at P14–P30 (Ivy and Scott, 2021), and if this effect persists into adulthood then it might have contributed to some of the plasticity in arterial O_2_ saturation observed here. Highlanders exhibit higher cardiac output (largely *via* increased stroke volume) at V̇O_2max_ than low-altitude deer mice and white-footed mice, and they increase cardiac output by a greater magnitude in response to hypoxia acclimation (Tate et al., 2020). Highlanders also exhibit greater capillarity, oxidative capacity, and mitochondrial density in the gastrocnemius (a large hindlimb muscle important for shivering thermogenesis) than lowlanders, but neither developmental hypoxia nor adult hypoxia acclimation affect these traits (Lui et al., 2015; Scott et al., 2015; Mahalingam et al., 2017, 2020; Nikel et al., 2018). Therefore, plasticity during development and adulthood along with evolved changes across the O_2_ pathway contribute to increasing thermogenic V̇O_2max_ in deer mice at high altitude.

Some other observed effects of hypoxia could represent maladaptive plasticity. Our finding that lowland white-footed mice exhibit right-ventricle hypertrophy in response to hypoxia acclimation in adulthood is consistent with recent findings in low-altitude deer mice and white-footed mice (Velotta et al., 2018; West et al., 2021b) along with many other lowland taxa (Monge-C et al., 1992; Zungu et al., 2008; Jochmans-Lemoine et al., 2015). This likely represents a pathological outcome of hypoxic pulmonary hypertension (HPH), a maladaptive response to chronic hypoxia that contributes to disease in humans (e.g., mountain sickness) and other mammals (e.g., brisket disease in cattle; Monge-C et al., 1992; Rhodes, 2005). We also found that parental hypoxia exposure in lowlanders led to right-ventricle hypertrophy in offspring that were never exposed to hypoxia themselves. In contrast, in high-altitude deer mice, HPH is attenuated and right-ventricle hypertrophy does not occur during hypoxia exposure in adulthood (Velotta et al., 2018; West et al., 2021b), and parental hypoxia does not lead to persistent effects on the right ventricle. The difference between high-altitude deer mice and white-footed mice in the effects of chronic hypoxia in adulthood on right-ventricle mass is associated with appreciable differences in right-ventricle gene expression (Velotta et al., 2018). However, developmental hypoxia did lead to right-ventricle hypertrophy, suggesting that high-altitude deer mice have not completely eliminated all of the detrimental effects of life-long exposure to chronic hypoxia.

Whether developmental plasticity has evolved in high-altitude deer mice remains an open question. We have previously shown that the hypoxia acclimation response of adults is augmented in highlanders, leading to exaggerated increases in thermogenic V̇O_2max_ as compared to lowland deer mice and white-footed mice (Tate et al., 2017, 2020). Our results here show that developmental hypoxia elicits even greater increases in thermogenic V̇O_2max_ in highlanders, but it is not yet clear whether the magnitude of this developmental response has evolved. On the one hand, developmental hypoxia was intolerable to lowland white-footed mice, which were unable to raise offspring to weaning in hypoxia, as previously observed in rats (Jochmans-Lemoine et al., 2015). Therefore, evolved characteristics of deer mice enabled developmental plasticity in hypoxia by supporting offspring survival. On the other hand, the evolved differences in cardiorespiratory physiology in highland parents likely reduced the level of hypoxia experienced by their offspring during prenatal development in utero. Differences in survival and the developmental hypoxia response might therefore be explained by different levels of fetal hypoxia exposure. Whether or not developmental plasticity has evolved, our findings show that it can lead to adaptive increases in thermogenic capacity that contributes to the success of deer mice in high-altitude environments.

## Data Availability Statement

The datasets presented in this study can be found in online repositories. The names of the repository/repositories and accession number(s) can be found below: http://dx.doi.org/10.17632/wy2xmwfc9d.1; Mendeley Data.

## Ethics Statement

The animal study was reviewed and approved by McMaster University Animal Research Ethics Board.

## Author Contributions

GS and CI designed the study. CI carried out mouse breeding, ran and analyzed the *in vivo* experiments. HP and CW carried out histological preparation and analysis. CI and GS wrote the manuscript, and all authors edited the manuscript. All authors contributed to the article and approved the submitted version.

## Conflict of Interest

The authors declare that the research was conducted in the absence of any commercial or financial relationships that could be construed as a potential conflict of interest.

## Publisher’s Note

All claims expressed in this article are solely those of the authors and do not necessarily represent those of their affiliated organizations, or those of the publisher, the editors and the reviewers. Any product that may be evaluated in this article, or claim that may be made by its manufacturer, is not guaranteed or endorsed by the publisher.
