# Supplementary material for: Distinct Mechanisms Underlie Developmental Plasticity and Adult Acclimation of Thermogenic Capacity in High-Altitude Deer Mice
Source: Front Physiol. 2021 Aug 11;12:718163. doi: 10.3389/fphys.2021.718163 (PMC8385410; doi:10.3389/fphys.2021.718163)
Supplement: Supplementary file 1 [file Data_Sheet_1.PDF]

SUPPLEMENTAL TABLES

Table S1. Results of linear mixed-effect models for ventilatory and metabolic variables during thermogenic $\dot{V}O_{2max}$ in 12 kPa O_2 and haematological measurements. Body mass was included as a covariate, and family and sex were included as random factors in initial models. NS, not significant and excluded from final model. See Methods for additional details on statistical testing.

Trait		Highlander				Lowlander			
		Treatment	Body mass	Family	Sex	Treatment	Body mass	Family	Sex
Thermogenic $\dot{V}O_{2max}$	F	6.043	32.23	-	-	7.338	18.95	-	-
	P	0.002	<0.001	NS	NS	0.011	<0.001	NS	NS
Total ventilation	F	12.59	-	-	-	18.07	2.786	-	-
	P	<0.001	NS	NS	0.040	<0.001	0.105	NS	NS
Arterial O_2 saturation	F	6.635	-	-	-	0.533	8.353	-	-
	P	0.001	NS	NS	NS	0.472	0.008	NS	NS
Heart rate	F	1.908	-	-	-	0.679	-	-	-
	P	0.147	NS	NS	NS	0.417	NS	NS	0.047
Breathing frequency	F	0.945	-	-	-	15.03	-	-	-
	P	0.428	NS	NS	NS	<0.001	NS	NS	NS
Tidal volume	F	9.476	-	-	-	0.008	-	-	-
	P	<0.001	NS	NS	0.010	0.977	NS	NS	NS
Air convection requirement	F	6.546	5.863	-	-	0.654	6.817	-	-
	P	0.001	0.020	NS	NS	0.425	0.014	NS	NS
Pulmonary O_2 extraction (%)	F	5.325	2.684	-	-	0.534	2.887	-	-
	P	0.004	0.109	NS	NS	0.470	0.099	NS	NS
Blood haemoglobin content	F	0.045	-	-	-	33.01	-	-	-
	P	0.987	NS	NS	NS	<.001	NS	NS	0.035
Haematocrit	F	21.44	-	-	-	65.00	-	-	-
	P	<0.001	NS	NS	NS	<0.001	NS	NS	NS
Mean corpuscular	F	4.164	-	-	-	0.651	12.38	-	-
haemoglobin content	P	0.012	NS	NS	NS	0.427	0.001	NS	0.034
Right ventricle mass relative to	F	6.841	-	-	-	23.61	3.553	-	-
left ventricle and septum mass	P	0.001	NS	NS	NS	<0.001	0.069	NS	NS

Table S2. Results of linear mixed-effect models for ventilatory and metabolic variables during thermogenic $\dot{V}O_{2max}$ in 21 kPa O_2. Body mass was included as a covariate, and family and sex were included as random factors in initial models. NS, not significant and excluded from final model. See Methods for additional details on statistical testing.

Trait		Highlander				Lowlander			
		Treatment	Body mass	Family	Sex	Treatment	Body mass	Family	Sex
Thermogenic $\dot{V}O_{2max}$	F	2.865	46.47	-	-	0.180	19.15	-	-
	P	0.049	<0.001	NS	NS	0.674	<0.001	NS	NS
Total ventilation	F	0.170	-	-	-	8.906	8.899	-	-
	P	0.916	NS	0.003	NS	0.006	0.006	NS	NS
Arterial O_2 saturation	F	0.735	4.215	-	-	0.033	4.357	-	-
	P	0.539	0.048	NS	NS	0.858	0.046	NS	NS
Heart rate	F	0.015	-	-	-	0.414	-	-	-
	P	0.997	NS	NS	NS	0.525	NS	NS	NS
Breathing frequency	F	1.521	-	-	-	4.868	-	-	-
	P	0.224	NS	NS	NS	0.035	NS	NS	NS
Tidal volume	F	0.404	-	-	-	0.208	4.408	-	-
	P	0.751	NS	0.013	NS	0.652	0.044	NS	NS
Air convection requirement	F	0.489	2.752	-	-	4.342	2.976	-	-
	P	0.692	0.105	0.019	NS	0.046	0.094	NS	NS
Pulmonary O_2 extraction (%)	F	5.954	-	-	-	4.530	9.404	-	-
	P	0.002	NS	0.003	NS	0.041	0.004	NS	NS

Table S3. Measurements at thermogenic $\dot{V}O_2$max in normoxia (21 kPa O_2) and hypoxia (12 kPa O_2) for highland deer mice and lowland white-footed mice exposed to parental hypoxia. Data are mean ± SEM followed by statistical main effects of parental hypoxia treatment. N= 7 for highlanders and 6 for lowlanders. * Significant pairwise difference from controls within a population.

	Highlander		Lowlander	
Trait	21 kPa O_2	12 kPa O_2	21 kPa O_2	12 kPa O_2
Thermogenic $\dot{V}O_{2max}$ (ml min^{-1} g^{-1})	0.229 ± 0.026 $F_{1,19}$=0.067, P=0.799	0.151 ± 0.013 $F_{1,19}$=0.002, P=0.962	0.181 ± 0.015 $F_{1,11}$=0.320, P=0.583	0.129 ± 0.006 $F_{1,11}$=2.011, P=0.184
Arterial O_2 saturation (%)	98.79 ± 0.51 $F_{1,19}$=0.001, P=0.994	90.03 ± 1.95* $F_{1,19}$=12.99, P=0.002	98.85 ± 0.25 $F_{1,11}$=2.165, P=0.175	73.97 ± 2.92 $F_{1,11}$=3.76, P=0.084
Heart rate (beats min^{-1})	591.8 ± 21.6 $F_{1,19}$=2.034, P=0.173	460.8 ± 42.9 $F_{1,19}$=1.634, P=0.218	616.1 ± 12.6 $F_{1,11}$=0.004, P=0.952	494.7 ± 39.8 $F_{1,11}$=0.562, P=0.471
Total ventilation (ml min^{-1} g^{-1})	5.05 ± 0.34 $F_{1,19}$=0.089, P=0.769	4.89 ± 0.61 $F_{1,19}$=0.206, P=0.655	4.17 ± 0.30 $F_{1,11}$=0.575, P=0.463	3.78 ± 0.33 $F_{1,11}$=1.099, P=0.317
Breathing frequency (min^{-1})	400.9 ± 28.7 $F_{1,19}$=0.024, P=0.878	347.7 ± 28.7 $F_{1,19}$=0.921, P=0.349	429.8 ± 19.7* $F_{1,11}$=5.643, P=0.035	365.0 ± 22.3 $F_{1,11}$=0.448, P=0.516
Tidal volume (μl g^{-1})	12.88 ± 0.99 $F_{1,19}$=0.098, P=0.757	14.13 ± 1.85 $F_{1,19}$=1.382, P=0.254	9.78 ± 0.71* $F_{1,11}$=5.245, P=0.041	10.44 ± 0.89* $F_{1,11}$=10.02, P=0.010
Air convection requirement (ml air ml O_2^{-1})	23.45 ± 2.52 $F_{1,19}$=0.062, P=0.806	32.39 ± 3.23 $F_{1,19}$=0.626, P=0.439	22.02 ± 0.45 $F_{1,11}$=0.581, P=0.461	29.11 ± 1.14* $F_{1,11}$=4.141, P=0.065
Pulmonary O_2 extraction (%)	23.78 ± 3.15 $F_{1,19}$=0.072, P=0.791	27.50 ± 2.94 $F_{1,19}$=0.001, P=0.974	22.17 ± 0.36 $F_{1,11}$=0.369, P=0.556	28.47 ± 1.11 $F_{1,11}$=4.111, P=0.068

Table S4. Measurements at thermogenic VO_{2max} in hypoxia (12 kPa O_2) and normoxia (21 kPa O_2) for highland deer mice and lowland white-footed mice chronically exposed to hypoxia at different stages of development. Data are mean ± SEM. For highlanders, N= 14 control, 12 adult hypoxia, 11 post-natal hypoxia, 7 life-long hypoxia; for lowlanders, N= 18 control, 16 adult hypoxia. *Significant pairwise difference from controls within a population.

Trait	Treatment	Highlander	Lowlander
Thermogenic VO_{2max} at 12 kPa O_2			
Breathing frequency (min^{-1})	Control	376.3 ± 15.6	341.5 ± 12.6
	Adult hypoxia	407.3 ± 18.0	411.8 ± 13.0*
	Post-natal hypoxia	411.4 ± 25.2	-
	Life-long hypoxia	415.4 ± 12.1	-
Air convection requirement	Control	30.73 ± 1.25	35.04 ± 1.55
(ml air ml O_2^{-1})	Adult hypoxia	29.61 ± 1.80	37.85 ± 2.47
	Post-natal hypoxia	38.44 ± 3.40	-
	Life-long hypoxia	46.40 ± 5.47*	-
Pulmonary O_2 extraction (%)	Control	27.58 ± 1.11	24.17 ± 0.98
	Adult hypoxia	29.07 ± 1.81	22.84 ± 1.18
	Post-natal hypoxia	23.09 ± 1.86	-
	Life-long hypoxia	19.45 ± 2.17*	-
Thermogenic VO_{2max} at 21 kPa O_2			
Breathing frequency (min^{-1})	Control	405.0 ± 12.1	365.1 ± 13.2
	Adult hypoxia	443.5 ± 19.2	404.0 ± 11.4*
	Post-natal hypoxia	396.3 ± 16.2	-
	Life-long hypoxia	401.3 ± 29.5	-
Air convection requirement	Control	24.93 ± 3.98	22.78 ± 0.95
(ml air ml O_2^{-1})	Adult hypoxia	20.53 ± 0.99	25.71 ± 0.90*
	Post-natal hypoxia	26.95 ± 2.80	-
	Life-long hypoxia	28.74 ± 2.32	-
Pulmonary O_2 extraction (%)	Control	24.24 ± 2.34	22.21 ± 0.92
	Adult hypoxia	25.04 ± 1.37	19.57 ± 0.70*
	Post-natal hypoxia	20.36 ± 1.60	-
	Life-long hypoxia	18.52 ± 1.35*	-

Table S5. Results of linear mixed-effect models for lung volume and histology measurements. Body mass was included as a covariate, and family and sex were included as random factors in initial models. NS, not significant and excluded from final model. See Methods for additional details on statistical testing.

Trait		Highlander				Lowlander			
		Treatment	Body mass	Family	Sex	Treatment	Body mass	Family	Sex
Body mass	F	0.027	-	-	-	0.642	-	-	-
	P	0.974	-	NS	NS	0.440	-	NS	NS
Lung volume	F	5.426	1.965	-	-	4.260	2.270	-	-
	P	0.028	0.194	NS	0.057	0.066	0.163	NS	NS
Mean alveolar	F	0.989	1.509	-	-	0.209	0.025	-	-
surface density	P	0.406	0.247	NS	NS	0.657	0.879	NS	NS
Total alveolar	F	1.467	1.650	-	-	4.162	1.429	-	-
surface area	P	0.276	0.228	NS	NS	0.069	0.260	NS	NS
Alveolar density	F	0.529	0.221	-	-	5.795	0.497	-	-
	P	0.608	0.651	0.014	NS	0.037	0.497	NS	NS

Table S6. Tissue measurements of parental hypoxia group for highland deer mice and lowland white-footed mice. Data are mean ± SEM, N as in Table S3, followed by statistical main effects of parental hypoxia treatment. * Significant difference from controls within a population

Trait	Highlander	Lowlander
Haemoglobin (g dl^{-1})	20.56 ± 1.65<br>$F_{1,19}$=0.628, P=0.440	30.04 ± 1.09*<br>$F_{1,11}$=179.6, P<0.001
Haematocrit (%)	46.99 ± 1.70<br>$F_{1,19}$=0.125, P=0.728	44.76 ± 0.77<br>$F_{1,11}$=0.181, P=0.679
Mean corpuscular haemoglobin concentration (g dl^{-1})	42.18 ± 3.70<br>$F_{1,19}$=0.133, P=0.720	67.26 ± 3.05*<br>$F_{1,11}$=168.8, P<0.001
Right ventricle mass relative to left ventricle and septum mass	0.20 ± 0.01<br>$F_{1,19}$=0.034, P=0.856	0.32 ± 0.02*<br>$F_{1,11}$=7.60, P=0.017
